# Mobile Applications for Longitudinal Data Collection: Web-based Survey Study of Former Intensive Care Patients

**DOI:** 10.1007/s10916-025-02151-w

**Published:** 2025-01-31

**Authors:** Denise Molinnus, Anne Mainz, Angelique Kurth, Volker Lowitsch, Matthias Nüchter, Frank Bloos, Thomas Wendt, Philipp Potratz, Gernot Marx, Sven Meister, Johannes Bickenbach

**Affiliations:** 1 Department of Intensive Care Medicine, Faculty of Medicine, RWTH Aachen University, Aachen, Germany; 2 Health Informatics, Faculty of Health/School of Medicine, Witten/Herdecke University, Witten, Germany; 3 Healthcare IT Solutions GmbH, Aachen, Germany; 4 LIFE Management Cluster, Medical Faculty, University of Leipzig, Leipzig, Germany; 5 Department of Anesthesiology and Intensive Care Medicine, Jena University Hospital, Jena, Germany; 6 Institute for Medical Informatics, Statistics and Epidemiology, University of Leipzig, Leipzig, Germany; 7 Center for Clinical Studies and Applied Healthcare Research, St. Francis Foundation Münster, Münster, Germany; 8 Department Healthcare, Fraunhofer Institute for Software and Systems Engineering ISST, Dortmund, Germany

**Keywords:** PICS, mHealth, Mobile Application, Digital Health, Digital Skills, ICU, Mobile Phone

## Abstract

Purpose: Mobile health plays an important role in providing individualized information about the health status of patients. Limited information exists on intensive care unit (ICU) patients with the risk of suffering from the post-intensive care syndrome (PICS), summarizing long-term physical, mental and cognitive impairment. This web-based survey study aims to identify specific needs of former ICU patients for utilizing a newly developed, so called Post-Intensive Care Outcome Surveillance (PICOS) app to collect relevant PICS-related parameters. Methods: A prototype app was developed following interaction principles for interactive systems of usability engineering. Patients from four different German hospitals were asked about demographics, interaction with technology and their perception of the prototype regarding hedonic motivation, perceived ease of use and performance expectancy. Results: 123 patients participated in the survey; the majority owned and used smartphones. Nearly half of respondents would seek help from family members or caregivers using the app. There was a difference in affinity for technology for participants who own a smartphone and those who do not, t(116) = − 0.97, *p* = .335, and no significant difference in affinity for technology whether the participants would like support when using the app or not, t(97) = 1.81, *p* = .073. The average hedonic motivation for using the app was M = 4.44 (SD = 1.304). Conclusion: This app prototype was perceived as both beneficial and easy to use, indicating its success among former ICU patients. Due to aging and ongoing health impairments, every second patient would need assistance with the initial use of the app.

**Supplementary Information:**

The online version contains supplementary material available at 10.1007/s10916-025-02151-w.

## Introduction

The collection and analysis of data from former intensive care patients is particularly interesting due to the currently limited information available about this patient population. Survival rates of patients treated in intensive care units (ICU) have increased worldwide in recent years [1, 2]. However, the period following a potentially protracted ICU stay is often characterized by a prolonged recovery process, including suffering from long-term physical, mental and cognitive impairments, a constellation of symptoms often referred to as post-intensive care syndrome (PICS) [3–7]. This term is used to describe the interaction of a broad range of disorders arising after ICU discharge that were not diagnosed before ICU treatment. The prevalence of the symptoms varies widely among different ICU patient groups with an increased risk of morbidity and mortality [8]. Furthermore, patients often need assistance in their daily life, which has a tremendous effect on the whole family life [9]. There is an urgent need to learn more about this particular patient group and potential long-term impairments.

In this context, the development of a user-friendly, easy-to-handle and easy-to-understand app, would be beneficial (a) for the collection of data relevant to research on PICS, (b) to support a more personalized treatment, and (c) for engaging patients in daily self-care routines to learn and understand more about their own health condition. mHealth apps have shown successful results for other diseases to facilitate patient engagement and collect information [10, 11]. Since PICS-patients often exhibit significant constraints and a heterogeneous age range, it is particularly important to ascertain in advance how the app should be precisely tailored to ensure that patients derive satisfaction and are inclined to use it regularly.

As part of the national Medical Informatics Initiative (MI-I), the DISTANCE project on which this research is based also strives to improve the availability of health-related data for research purposes. The prioritized aim of this research therefore was to identify the needs of former ICU patients for the utilization of an app to foster (a) and the potential for later extension to (b) and (c).

The following objectives were addressed:

O1: Development of a prototype of a patient-centred app, called the PICOS app (Post-Intensive Care Outcome Surveillance).

O2: Presentation of O1 to former intensive care patients to answer the hypotheses H1 to H3 defined in chapter “Hypotheses”.

## Methods

The web-based survey was conducted as a multi-centre quality assurance measure and quantitative data from potential users were collected as an empirical evaluation procedure. The reporting complied with the CONSORT-EHEALTH checklist (V.1.6.1) by Eysenbach [12] for evaluation reports of web-based and mobile health interventions.

### Study Use Case

The study-related use case focuses on the aim of collecting PICS-relevant data for research through the app to be designed in this paper. As part of the national MI-I, this means that data is to be described using international standards (in particular HL7 FHIR) and managed via so-called data integration centers in order to make it reusable for research. Integration into hospital-specific electronic health records or the national electronic patient record is not intended currently. The sole purpose is data collection for research.

In the future, after the patient’s discharge, various parameters (see below) will be entered by the patient himself on his mobile device using the PICOS app, either daily or weekly. This data is transmitted to the data integration centre of the project-specific node of the MI-I. In addition, it is conceivable that the use case could be expanded in the future to include the research findings of this study to support healthcare provision.

### Recruitment

Potential future app users over 18 years old were recruited to take part in this survey study. Patients, who had been treated in the ICU for at least 72 h or who had been mechanically ventilated for more than 24 h were eligible to participate. As the aim of the survey is a potential impairment of PICS in former intensive care patients, participants were screened as soon as they met the criteria for potential ICU discharge and were then included upon transfer to the general ward. Pregnant women (due to potentially redundant hospital stay due to other reasons) and patients who were unable complete the questionnaire themselves were excluded. Study nurses approached potential participants on the general ward shortly after their transfer from the intensive care unit to enrol them into the survey study, just as it will be when patients use the PICOS app. The future aim is to ensure that patients can begin using the app while still hospitalized on the general ward, allowing project staff to be readily available for any questions or assistance.The questionnaire was written in German. Patients were recruited from four different hospitals in Germany: University Hospital RWTH Aachen, University Hospital Jena, University Hospital Leipzig and St. Franziskus Hospital Münster.

### Prototype of the PICOS app

A clickable prototype of the developed PICOS app created using the prototyping tool *Adobe XD*. The prototype served to make the planned design tangible and give potential users an impression of the planned functionality and design. The prototype included a main page where the users were able to track their registered vital signs like blood glucose level, heart rate, blood pressure and weight as well as their activity through step tracking, with manual entries or via wearables (e.g. smartwatch). A weekly use of the PHQ-4 questionnaire (patient health questionnaire-4) [13] with a 4-item scale asking for depression symptoms and anxiety was also was also incorporated into the app. No real data were captured when using this prototype.

**Fig. 1:**
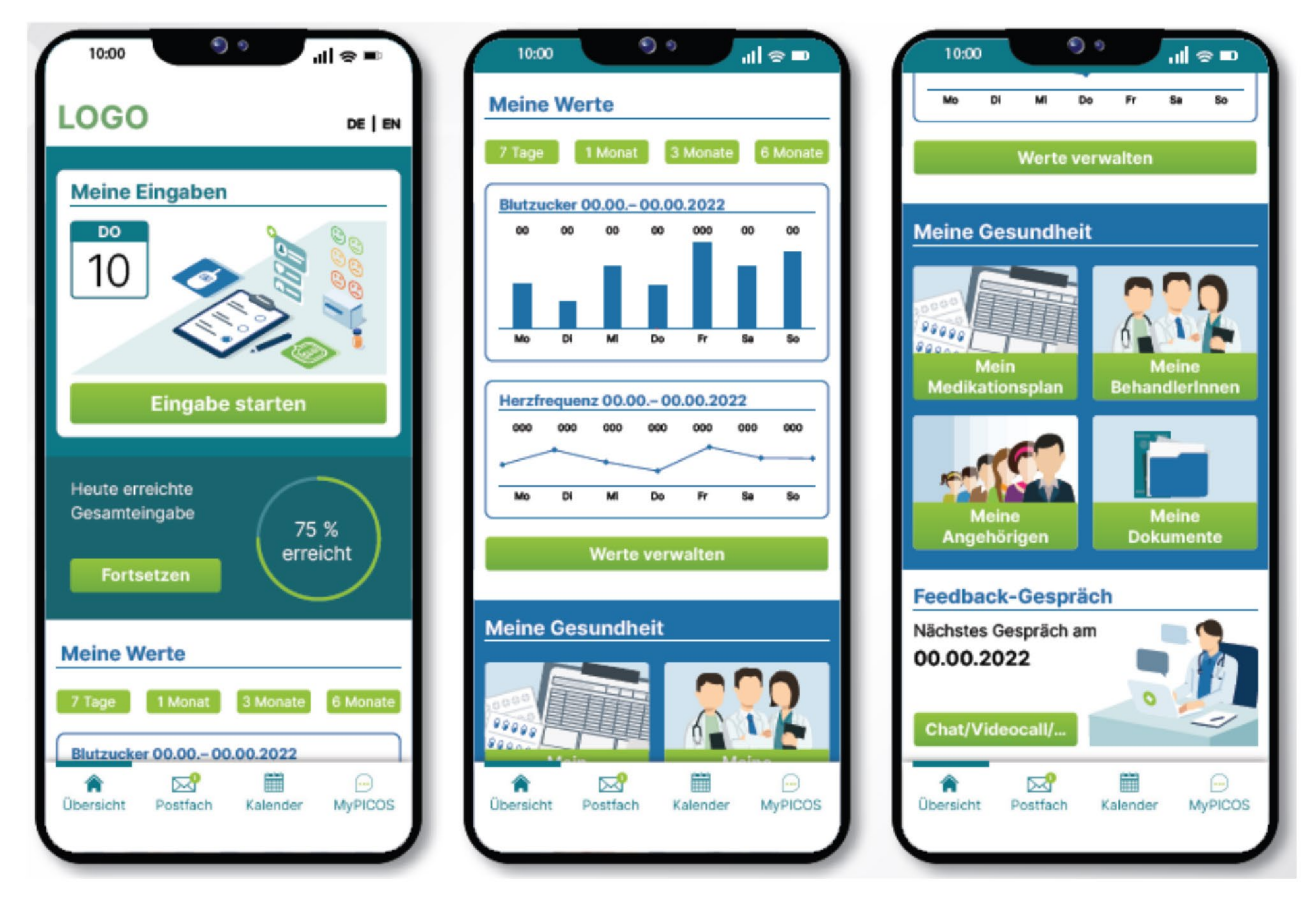
Screenshots of the PICOS clickable prototype, German version Left: Main page where users can register their vital signs and activity, Middle: Graphical overview of blood sugar and heart rate over one week, Right: Access to store information such as medication plan, contacts of treating physicians/caregivers and relatives and additional documents as well as an appointment reminder

In the PICOS app, the users receive daily reminders about the completeness of their entries. From the main page, users can access an interface to manage their vital signs directly, update them or indicate if they are not relevant. From the main page, users could also access their medication plan, view their therapy plan, create an overview of caregivers and relatives, save documents, or manage their doctor and hospital visits (see Fig. 1).

The main goals of the design were to create a usable interface with simple and intuitive operations. The user interface followed the principles of usability engineering for interactive systems as formulated in ISO 9241 − 110 as an international standard [14]. The following principles were considered: task appropriateness, self-descriptiveness, conformity to expectations, learnability, controllability, fault tolerance, customizability.

### Questionnaires for Evaluation

The questionnaires for evaluating the PICOS app prototype covered the demographic data of the participants, their interaction with technology and their perception of the prototype. Due to the vulnerability of the target group, the survey was kept as short as possible. The survey was evaluated by an expert panel with four researchers following the recommendations of Ikart [15]. All items used had already been validated and applied in other surveys.

For demographics, participants were asked about their age, their education level and gender identity. In addition, participants were asked if they own or use a smartphone and whether they would like support (e.g., from family members or caregivers) when using the PICOS app.

The interaction with technology was operationalised by the German version of the *Affinity for Technology Interaction (ATI)* scale developed by Franke et al. [16] and based on the need for cognition-theory [17].

The perception of the PICOS app was measured using the scale by Krishnan et al. [18] which addresses the intention to use CHI (consumer health informatics) applications, based on [19,20, 21]. Because representatives of the target group quickly showed signs of exhaustion during the pre-testing, the number of items had to be reduced by the expert panel to: h*edonic motivation*, *perceived ease of use* and *performance expectancy*. These dimensions showed a significant linear relationship with the adoption behaviour of CHI-Applications [18, 22–25], so we interpret the overall value for all these dimensions as the intention to use the PICOS app.

### Hypotheses

The questionnaires used are intended to analyse the characteristics of the target group and the relationship between the following characteristics:

#### H1

There is a significant relationship between the affinity for technology interaction and.

- …the age of the participants.
- …the gender identity of the participants.
- …the possession and usage of a smartphone.
- …the interest in support when using the PICOS app.

#### H2

There is a significant relationship between the perception of the PICOS app and.

- …the age of the participants.
- …the gender identity of the participants.
- …the possession and usage of a smartphone.
- …the interest in support when using the PICOS app.

#### H3

: There is a significant relationship between the affinity for technology interaction and the perception of the PICOS app.

### Procedure

The web-based survey was conducted between 01.09.2022 and 01.09.2023. It was implemented as an online survey via LimeSurvey, but participants completed the questionnaires with the help of a study nurse. The entire survey took no longer than 15 min.

### Statistical Analysis

The collected data were analysed with the IBM SPSS Statistics 28 analysis software and analysed descriptively. Normal distribution was tested by the Shapiro-Wilk test [26]. Correlations were tested through Spearman’s ρ for ordinal variables or rejected normal distribution. For evaluation of the Spearman’s ρ effect sizes, the classification according to Cohen [27] was chosen with 0.10–0.29 as a small/weak effect, 0.30–0.49 as a medium/moderate effect, and > = 0.50 as a large/strong effect. Levene’s Test was used to test for homogeneity of variances with a significance level of 0.05.

## Results

### Participants

A total of *N* = 123 participants took part in the evaluation. Of these, *n* = 30 (24.4%) of them identified as female, and *n* = 93 (75.6%) as male. Most participants were between 50 and 65 years old (*n* = 57; 46.3%). The most common education level was primary or lower secondary education (*n* = 37; 30.1%) (see Table 1).

**Table 1:** Age group and education level of the participants

Age group	*n*	percent
Between 18 and 33 years	10	8.1%
Between 34 and 49 years	9	7.3%
Between 50 and 65 years	57	46.3%
Between 66 and 81 years	41	33.3%
Between 82 and 97 years	6	4.9%
Education level	*n*	percent
Primary education/ Lower secondary education	37	30.1%
Intermediate/ General secondary education	34	27.6%
General or subject-restricted higher education entrance qualification	23	18.7%
Bachelor’s degree	6	4.9%
Master’s degree	20	16.3%
Doctorate	3	2.4%

### Interaction with Technology

The majority of the participants, 92.7% (*n* = 114), reported owning and using their own smartphone, while 7.3% (*n* = 9) participants did not own or use such a device. About half of the potential users (*n* = 48; 49.5%) would like to have support when using the app.

The mean *affinity for technology interaction* was *M* = 3.77 (*SD* = 1.232). Age and *affinity for technology interaction* had a significant (*p* < .05) but weak negative correlation, Spearman’s ρ = − 0.199; *p* = .03. When comparing the data for different gender, there was a statistically significant difference (*p* < .05) in the *affinity for technology interaction*, *t(116)* = 2.36, *p* = .020. Male participants had a significantly higher *affinity for technology interaction* (*M* = 3.87, *SD* = 1.25) than female participants (*M* = 3.27, *SD* = 1.06). There was no statistically significant difference in affinity for technology for participants who own a smartphone and those who do not, *t*(116) = − 0.97, *p* = .335, nor in whether the participants would like support when using the app, *t*(97) = 1.81, *p* = .073.

### Perception of the app Prototype

The average *hedonic motivation* for using the app was *M* = 4.44 (*SD* = 1.304), *perceived ease of use M* = 5.53 (*SD* = 1.415) and *performance expectancy M* = 5.21 (*SD* = 1.392). The sum of all these factors resulted in the *intention to use the app* with an average value of *M* = 4.87 (*SD* = 1.042). An overview is given in Table 2.

**Table 2:** Intention to use and perception of the PICOS app

	1 (completely disagree)	2	3	4	5	6	7 (completely agree)	Mean	Median	SD
*Hedonic motivation*										
	1.7%	7.8%	9.5%	37.9%	22.4%	14.7%	6.0%	4.44	4	1.304
*Perceived ease of use*										
	0%	4.3%	4.3%	8.7%	17.4%	33.9%	31.3%	5.53	6	1.415
*Performance expectancy*										
	0.9%	6.1%	4.3%	17.4%	22.6%	31.3%	17.4%	5.21	5	1.392
Intention to use the PICOS app (overall value)										
	0%	4.3%	6.1%	16.5%	33.0%	33.9%	6.1%	4.87	5	1.042

### Age Effects

Age and *hedonic motivation* had no significant correlation, Spearman’s ρ = − 0.044, *p* = .647, as well as age and *performance expectancy*, ρ = − 0.059, *p* = .531. Age and *perceived ease of use* correlated negatively with a medium effect, ρ = − 0.324, *p* = < 0.001 and age and *intention to use* correlated negatively with a small effect, ρ = − 0.205, *p* = .032.

### Gender Effects

There was no statistically significant difference in *hedonic motivation* for different genders, *t*(110) = -1.33, *p* = .185, *perceived ease of use*, *t*(115) = 0.57, *p* = .569, *performance expectancy*, *t*(115) = − 0.97, *p* = .333, or the *overall intention to use*, *t*(108) = -1.31, *p* = .192.

### Effects of Smartphone use

For *hedonic motivation*, *performance expectancy* and *overall intention to use*, no significant differences between the group owning and using a smartphone and the group not owning or using a smartphone not could be found (*t*_Hedonic motivation_(110) = − 0.27, *p =* .786; *t*_Performance expectancy_(115) = -1.35, *p =* .181; *t*_Intention to use_ (108) = -1.85, *p =* .067). *Perceived ease of use* had significant group differences for owning and using a smartphone or not, *t*(115) = -3.49, *p* = < 0.001. When owning and using a smartphone, participants perceived the ease of use of the app as higher (*M* = 5.64, *SD* = 1.32) than participants who did not own or use a smartphone (*M* = 3.92, *SD* = 1.78).

### Support Effects

No significant group differences between seeking support or not could be found for most of the different perception scores (*t*_*hedonic motivation*_(74.87) = -0.61, *p* = .541; *t*_Performance expectancy_(97) = 0.18, *p =* .858; *t*_Intention to use_ (92) = 1.13, *p =* .263). Only for *perceived ease of use*, there was a statistically significant difference between the seeking and not-seeking support group, *t*(98) = 2.61, *p* = .011. Participants who would not seek support perceived the ease of use significantly higher (*M* = 5.88; *SD* = 1.39) than participants who would seek support (*M* = 5.13; *SD* = 1.49).

### Affinity Effects

*Affinity for technical interaction* had a significant relationship *with* all dimensions of the perception of the PICOS app. *Affinity for technical interaction* and *hedonic motivation* correlated positively with a small effect, Spearman’s ρ = 0.282; *p* = .003. *Affinity for technical interaction* and *perceived ease of use* correlated positively with a medium effect, ρ = 0.392; *p* < .001, and *affinity for technical interaction* and *performance expectancy* correlated positively with a small effect, ρ = 0.279; *p* = .003. The overall *intention to use the PICOS app* and *affinity for technical interaction* correlated moderately, ρ = 0.359; *p* = .001, as *shown* in Table 3.

**Table 3:** Correlations with confidence intervals

Variable	1	2	3	4	5
*1. Age*					
*2. Affinity for technology*	− 0.199*				
*3. Hedonic motivation*	− 0.044	0.282**			
*4. Perceived ease of use*	− 0.324**	0.392**	0.431**		
*5. Performance Expectancy*	− 0.059	0.279**	0.763**	0.552**	
*6. Intention to use*	− 0.205*	0.359**	0.883**	0.759**	0.891**

* indicates *p* < .05, ** indicates *p* < .01

## Discussion

### Principal Findings

The main finding of this survey was that potential users assessed the PICOS app as beneficial and usable. Men showed a significantly higher affinity than women and the affinity for technical systems decreased significantly with age, which confirms hypotheses H1a and H1b. Surprisingly, people who owned a smartphone had no higher affinity for technology interaction than people who did not. There were also no differences in technical affinity for participants that would seek help and those who do not, therefore hypotheses H1c and H1d were rejected.

The participants evaluated the hedonic motivation of the clickable PICOS prototype on a medium level. Meaning they neither agreed nor disagreed on perceiving the app as fun, entertaining, enjoyable, pleasuring, exciting, thrilling or delightful. This assessment was robust for all characteristics analysed (gender, age, possession of smartphone, need of support). Factors that could increase hedonic motivation for mHealth applications would include the absence of app dysfunctions, variety in app features to avoid boredom, a number of useful features, appealing designs and no app dependence on internet, stated by Woldeyohannes and Ngwenyama [28]. However, the same authors questioned the significance of hedonic motivation for an mHealth app: “You won’t use an app for fun” [28]. Tamilmani et al. [29] went even further, doubting whether hedonic motivation is an appropriate construct when dealing with technology that has utilitarian purpose.

The participants tended to agree on the usefulness of the app for their daily life and health, this was also a robust result for all considered variables and underscores the usefulness of the app in the future beyond the collection of research data. Perceived usefulness is one of the key constructs of technology acceptance [20] and reflects also trust in the system and the entities behind it to achieve the desired outcomes enabling new possibilities or improving existing processes to achieve the desired outcomes through the app [30].The participants perceived the ease of use of the app during the learning process and during the daily interaction as rather easy. With older age, no smartphone ownership or use and the stated need for support among the patients, the perceived ease of use dropped significantly. Research shows that particularly middle aged and older adults – who make up the majority of the potential user group for the PICOS app – tend to give a higher value to the perceived ease of use when adopting a new digital application [31]. In total, hypotheses H2a, H2c and H2d were partially confirmed, H2b was rejected. When participants had a higher affinity for technical interaction, their perception of the app was significantly higher for all perception variables of the PICOS app, which confirms hypothesis H3.

When comparing the results with other studies [16] the ATI score of the participants was slightly above those of two samples of the general public in Germany. Regarding mHealth adoption in general, Uncovska et al. [32, 33] pointed out that perceived benefits of mHealth apps are more relevant than scientific evidence and an effortless usage had to be ensured. Multiple systematic reviews or meta-analyses showed that especially high levels of performance expectancy and perceived ease of use (or effort expectancy) had a strong effect on technology acceptance and use intentions of mHealth apps [34–36].

So far, there are few instruments to measure potential outcome parameters of PICS [37]. However, most of these instruments are only partly validated on ICU patients and there is also still shortage of tools measuring cognitive or physical activity. Furthermore, this data must be collected regularly and in large quantities to gain insights both for individual patients and for research. Based on existing tests designed to capture limitations in former intensive care patients, elements were integrated into the PICOS app. Using this app has the potential to streamline the retrieval and accessibility of medical information and data, thereby contributing to more efficient and effective healthcare services.

### Implementation of the PICOS app and the PICOS Ecosystem

Based on the findings of this study, the PICOS app for the Android operating system was implemented. It can be obtained for study participants via the Google app store. In addition, a separate web module was developed to enable medical staff to add data from the clinical context to the PICOS research dataset. The aim is to carry out a multicenter study for the clinical evaluation of the entire system.

### Limitations

A limitation of the survey is that the patients could only get a brief glimpse of the app and could only test it as a clickable prototype without real functionality. The impression in this test may differ from an actual use of the final app over a longer period. In addition, a recruitment bias can be assumed as particularly frail representatives of the target group either refused to take part in the study themselves or were excluded due to the conditions of participation. However, it can be assumed that people who did not participate in this study due to their very poor state of health would also not consider using the app. Therefore, the participants of this survey represent the potential user group well, but not necessarily the entire group of people who were treated on ICU. Another limitation is the timing of the survey. Patients were still in the hospital and have not yet fully recovered from their critical illness. Transferring the results to other user groups should also be done with caution, given the possible symptomatic impairments of patients in different areas of life [7]. Other target groups for similar mHealth apps, such as those without an ICU stay, may yield very different results. The following should also be noted when interpreting the results: As described within the technology acceptance model (TAM 3, Venkatesh and Bala [38]) and the Unified Theory of Acceptance and Use of Technology (UTAUT, Venkatesh et al. [39]) usage behavior is not the same as behavioral intention and only a result of the latter. This study therefore only makes statements regarding the behavioural intention, and conclusions regarding actual behaviour can only be drawn to a limited extent.

## Conclusion

This web-based survey study highlighted the initial phase of developing a user-friendly and intuitive app. Future studies should focus on the deployment of the developed PICOS app, enabling former intensive care patients to seamlessly integrate the app into their daily lives, starting directly after transferring from the ICU to the general ward to collect the initial data while still in the hospital and simultaneously provide on-site support for using the app. This integration enables the collection and storage of data trajectories post-hospital discharge, subsequently allowing scientists to analyse the data.

## Electronic Supplementary Material

Below is the link to the electronic supplementary material.

Supplementary Material 1

Supplementary Material 2

Supplementary Material 3

## Data Availability

Upon reasonable demand, anonymised data of the statistical calculation can be requested. The raw data cannot be provided due to the legal requirements of the ethics committee.

## Abbreviations

ATI: Affinity for Technology Interaction
HRQoL: Health related Quality of Life
ICU: Intensive Care Unit
PICOS: Post-Intensive Care Outcome Surveillance
PICS: Post-Intensive Care Syndrome
