# Supplementary material for: Mobile Applications for Longitudinal Data Collection: Web-based Survey Study of Former Intensive Care Patients
Source: J Med Syst. 2025 Jan 31;49(1):18. doi: 10.1007/s10916-025-02151-w (PMC11785681; doi:10.1007/s10916-025-02151-w)
Supplement: Supplementary file 2 — Supplementary Material 2 [file 10916_2025_2151_MOESM2_ESM.pdf]

Fragebogen PICOSVersion 3.0

Das hier vorgestellte Instrumentarium dient der Erhebung an Patient:innen des Projekts DISTANCE zur Bewertung der Intention zur Nutzung der sog. PICOS App. Das Instrumentarium ist hierzu in die folgenden Phasen unterteilt:

Demografie

Beantworten Sie bitte die nachfolgenden Fragen zu Ihrer Person. Kreuzen Sie die auf Sie zutreffende Kategorie an:

1. Wie alt sind Sie?

	Zwischen 18 und 33 Jahren
	Zwischen 34 und 49 Jahren
	Zwischen 50 und 65 Jahren
	Zwischen 66 und 81 Jahren
	Zwischen 82 und 97 Jahren
	Älter als 98
	Keine Angabe

2. Welchen höchsten Bildungsabschluss haben Sie?

	Hauptschulabschluss, Volksschulabschluss
	Realschulabschluss, Mittlere Reife, Polytechnische Oberschule
	Allgemeine oder fachgebundene Hochschulreife, Abitur
	Bachelor an (Fach-)Hochschule
	(Fach-) Hochschulabschluss (z.B. Diplom, Master, Magister, Staatsexamen)
	Promotion
	(noch) keinen Schulabschluss
	Keine Angabe

3. Welchem Geschlecht fühlen sie sich zugehörig?

	Männlich
	Weiblich
	Divers
	Keine Angabe

Technikinteraktion

Im Folgenden geht es um Ihre Interaktion mit technischen Systemen. Mit „technischen Systemen“ sind sowohl Apps und andere Software-Anwendungen als auch komplette digitale Geräte (z.B. Handy, Computer, Fernseher, Auto-Navigation) gemeint.

1. Bitte geben Sie den Grad Ihrer **Zustimmung** zu folgenden Aussagen an.

	Stimmt gar nicht	Stimmt weit-gehend nicht	Stimmt eher nicht	Stimmt eher	Stimmt weit-gehend	Stimmt völlig
1. Ich beschäftige mich gern genauer mit technischen Systemen.	☐	☐	☐	☐	☐	☐
2. Ich probiere gerne die Funktionen neuer technischer Systeme aus.	☐	☐	☐	☐	☐	☐
3. In erster Linie beschäftige ich mich mit technischen Systemen, weil ich muss.	☐	☐	☐	☐	☐	☐
4. Wenn ich ein neues technisches System vor mir habe, probiere ich es intensiv aus.	☐	☐	☐	☐	☐	☐
5. Ich verbringe sehr gern Zeit mit dem Kennenlernen eines neuen technischen Systems.	☐	☐	☐	☐	☐	☐
6. Es genügt mir, dass ein technisches System funktioniert, mir ist egal, wie oder warum.	☐	☐	☐	☐	☐	☐
7. Ich versuche zu verstehen, wie ein technisches System genau funktioniert.	☐	☐	☐	☐	☐	☐
8. Es genügt mir, die Grundfunktion eines technischen Systems zu kennen.	☐	☐	☐	☐	☐	☐
9. Ich versuche, die Möglichkeit eines technischen Systems vollständig auszunutzen.	☐	☐	☐	☐	☐	☐

Handynutzung

In diesem Abschnitt geht es um Ihre Smartphone Nutzungsgewohnheiten.

1. **Besitzen** und **Nutzen** Sie ein eigenes Smartphone? Bitte kreuzen Sie die zutreffende Antwort an.

	Ja
	Nein

App-Nutzung

Im Folgenden geht es um Ihre **Wahrnehmung** der im Projekt entwickelten **PICOS-App**. Bitte kreuzen Sie die für Sie zutreffenden Aussagen an. (Proband:innen testen einen Prototypen während der Studie. Screenshots dienen der Illustration.)

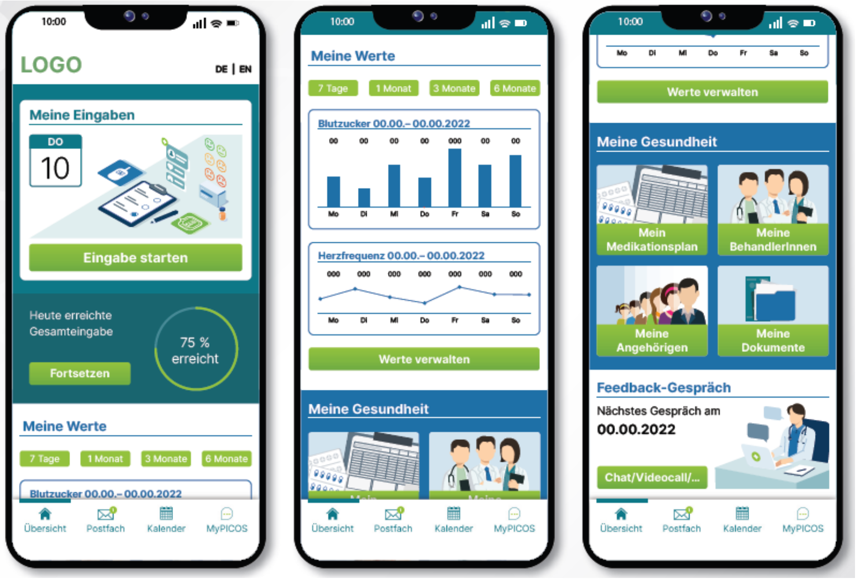

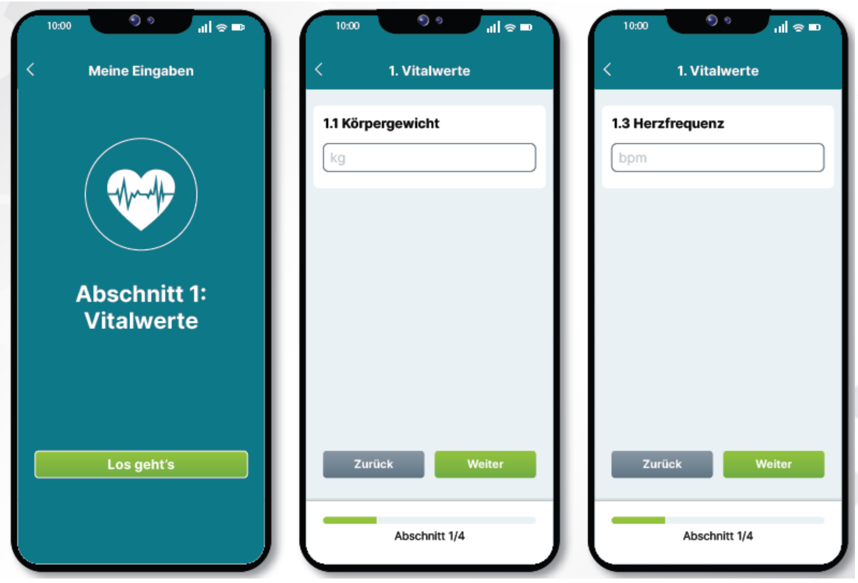

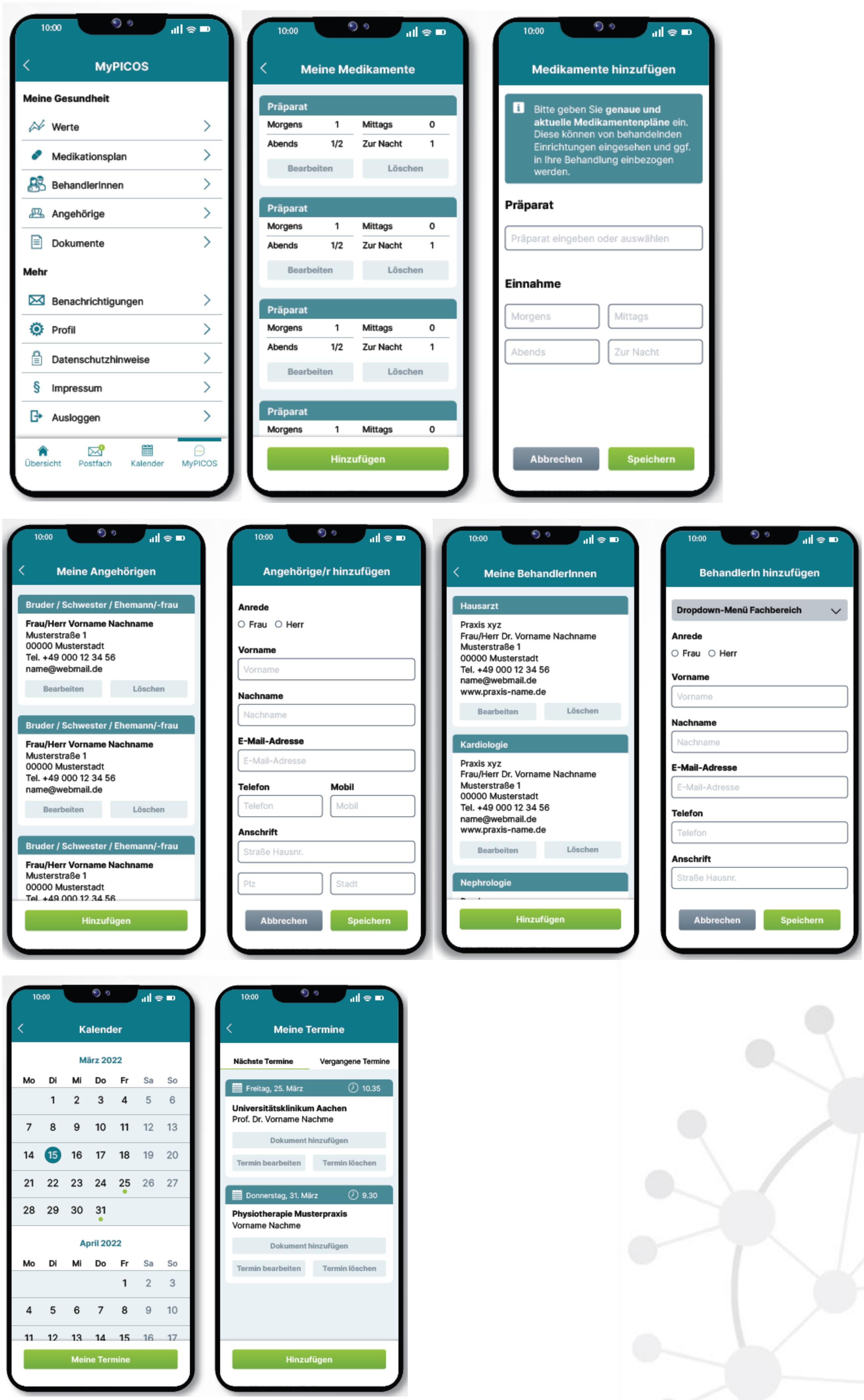
MyPICOS
Meine Gesundheit
Werte
Medikationsplan
BehandlerInnen
Angehörige
Dokumente
Mehr
Benachrichtigungen
Profil
Datenschutzhinweise
Impressum
Ausloggen
Übersicht
Postfach
Kalender
MyPICOS
Meine Medikamente
Präparat
Morgens 1 Mittags 0
Abends 1/2 Zur Nacht 1
Bearbeiten
Löschen
Hinzufügen
Medikamente hinzufügen
Bitte geben Sie genaue und aktuelle Medikamentenpläne ein. Diese können von behandelnden Einrichtungen eingesehen und ggf. in Ihre Behandlung einbezogen werden.
Präparat
Präparat eingeben oder auswählen
Einnahme
Morgens
Mittags
Abends
Zur Nacht
Abbrechen
Speichern
Meine Angehörigen
Bruder / Schwester / Ehemann/-frau
Frau/Herr Vorname Nachname
Musterstraße 1
00000 Musterstadt
Tel. +49 000 12 34 56
name@webmail.de
Bearbeiten
Löschen
Hinzufügen
Angehörige/r hinzufügen
Anrede
Frau
Herr
Vorname
Nachname
E-Mail-Adresse
Telefon
Mobil
Anschrift
Straße Hausnr.
Plz
Stadt
Abbrechen
Speichern
Meine BehandlerInnen
Hausarzt
Praxis xyz
Frau/Herr Dr. Vorname Nachname
Musterstraße 1
00000 Musterstadt
Tel. +49 000 12 34 56
name@webmail.de
www.praxis-name.de
Bearbeiten
Löschen
Kardiologie
Nephrologie
Hinzufügen
BehandlerIn hinzufügen
Dropdown-Menü Fachbereich
Anrede
Frau
Herr
Vorname
Nachname
E-Mail-Adresse
Telefon
Anschrift
Straße Hausnr.
Abbrechen
Speichern
Kalender
März 2022
Mo Di Mi Do Fr Sa So
April 2022
Meine Termine
Meine Termine
Nächste Termine
Vergangene Termine
Freitag, 25. März
10.35
Universitätsklinikum Aachen
Prof. Dr. Vorname Nachme
Dokument hinzufügen
Termin bearbeiten
Termin löschen
Donnerstag, 31. März
9.30
Physiotherapie Musterpraxis
Vorname Nachme
Hinzufügen

1. Würden Sie die Unterstützung eines Angehörigen, Betreuers o.ä. für die Nutzung der PICOS-App in Anspruch nehmen?

	Ja
	Nein

2. Bitte geben Sie den Grad Ihrer **Zustimmung** zu folgenden Aussagen an.

	Stimme überhaupt nicht zu	Stimme nicht zu	Stimme eher nicht zu	Stimme weder zu noch lehne ab	Stimme eher zu	Stimme zu	Stimme vollkommen zu
1. Die Verwendung der PICOS-App wird Spaß machen.	☐	☐	☐	☐	☐	☐	☐
2. Die Verwendung der PICOS-App wird unterhaltsam sein.	☐	☐	☐	☐	☐	☐	☐
3. Die Verwendung der PICOS-App wird angenehm sein	☐	☐	☐	☐	☐	☐	☐
4. Die Nutzung der PICOS-App wird mir Freude bereiten.	☐	☐	☐	☐	☐	☐	☐
5. Die Nutzung der PICOS-App wird aufregend sein.	☐	☐	☐	☐	☐	☐	☐
6. Die Nutzung der PICOS-App wird spannend sein.	☐	☐	☐	☐	☐	☐	☐
7. Die Verwendung der PICOS-App wird reizvoll sein.	☐	☐	☐	☐	☐	☐	☐
8. Das Erlernen der Nutzung der PICOS-App wird für mich einfach sein.	☐	☐	☐	☐	☐	☐	☐

9. Meine Interaktion mit der PICOS-App wird einfach sein.	☐	☐	☐	☐	☐	☐	☐
10. Die Interaktion mit der PICOS-App ist für mich klar und verständlich.	☐	☐	☐	☐	☐	☐	☐
11. Es wird mir leicht fallen, die PICOS-App zu beherrschen.	☐	☐	☐	☐	☐	☐	☐
12. Es wird mir leicht fallen, mich daran zu erinnern, wie ich die PICOS-App benutze.	☐	☐	☐	☐	☐	☐	☐
13. Insgesamt gehe ich davon aus, dass die PICOS-App einfach zu bedienen ist.	☐	☐	☐	☐	☐	☐	☐
14. Ich werde die PICOS-App in meinem täglichen Leben nützlich finden.	☐	☐	☐	☐	☐	☐	☐
15. Die Verwendung der PICOS-App kann meine Chancen auf eine bessere Gesundheit erhöhen.	☐	☐	☐	☐	☐	☐	☐
16. Die Verwendung der PICOS-App hilft mir, meine Gesundheit effizienter zu verwalten (zu überwachen).	☐	☐	☐	☐	☐	☐	☐
17. Die Nutzung der PICOS-App erhöht meine Fähigkeit, meine Gesundheit selbst in die Hand zu nehmen.	☐	☐	☐	☐	☐	☐	☐
18. Insgesamt empfinde ich die PICOS-App als vorteilhaft.	☐	☐	☐	☐	☐	☐	☐
19. Die Verwendung der PICOS-App für das Gesundheitsselbst-management würde mich sehr nervös machen.	☐	☐	☐	☐	☐	☐	☐
