# Supplementary material for: Mobile Applications for Longitudinal Data Collection: Web-based Survey Study of Former Intensive Care Patients
Source: J Med Syst. 2025 Jan 31;49(1):18. doi: 10.1007/s10916-025-02151-w (PMC11785681; doi:10.1007/s10916-025-02151-w)
Supplement: Supplementary file 3 — Supplementary Material 3 [file 10916_2025_2151_MOESM3_ESM.pdf]

PICOS Survey
Version 3.0

The instrument presented here is used in the DISTANCE project to assess (former) ICU-patients' intention to use the PICOS app. The instrument is divided into the following phases:

Participant Characteristics

Please answer the following questions about yourself. Tick the category that applies to you:

1. How old are you?

	Between 18 and 33 years
	Between 34 and 49 years
	Between 50 and 65 years
	Between 66 and 81 years
	Between 82 and 97 years
	Older than 98 years
	Prefer not to say

2. Which is your education level?

	Primary education/ Lower secondary education
	Intermediate/ General secondary education
	General or subject-restricted higher education entrance qualification
	Bachelor's degree
	Master's degree
	Doctorate
	no school-leaving certificate (yet)
	Prefer not to say

3. 3. Which gender do you identify as?

	Male
	Female
	Diverse
	Prefer not to say

Technology Interaction

In the following questionnaire, we will ask you about your interaction with technical systems. The term ‘technical systems’ refers to apps and other software applications, as well as entire digital devices (e.g. mobile phone, computer, TV, car navigation).

1. Please indicate the degree to which you agree/disagree with the following statements.

	completely disagree	largely disagree	slightly disagree	slightly agree	largely agree	completely agree
1. I like to occupy myself in greater detail with technical systems.	☐	☐	☐	☐	☐	☐
2. I like testing the functions of new technical systems.	☐	☐	☐	☐	☐	☐
3. I predominantly deal with technical systems because I have to.	☐	☐	☐	☐	☐	☐
4. When I have a new technical system in front of me, I try it out intensively.	☐	☐	☐	☐	☐	☐
5. I enjoy spending time becoming acquainted with a new technical system.	☐	☐	☐	☐	☐	☐
6. It is enough for me that a technical system works; I don’t care how or why.	☐	☐	☐	☐	☐	☐
7. I try to understand how a technical system exactly works.	☐	☐	☐	☐	☐	☐
8. It is enough for me to know the basic functions of a technical system.	☐	☐	☐	☐	☐	☐
9. I try to make full use of the capabilities of a technical system.	☐	☐	☐	☐	☐	☐

Smartphone Use

The following section is about your smartphone usage habits.

1. Do you **own and use** your own smartphone? Please tick the appropriate answer.

	Yes
	No

App Use

The following is about your perception of the PICOS app developed in the project. Please tick the statements that apply to you. (Test subjects can try out a click dummy during the study. Screenshots are used here for illustration purposes.)

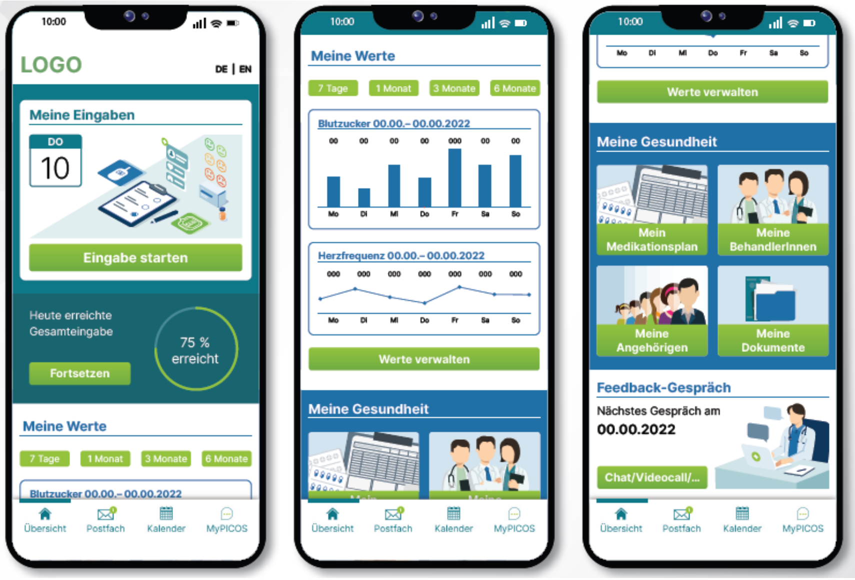

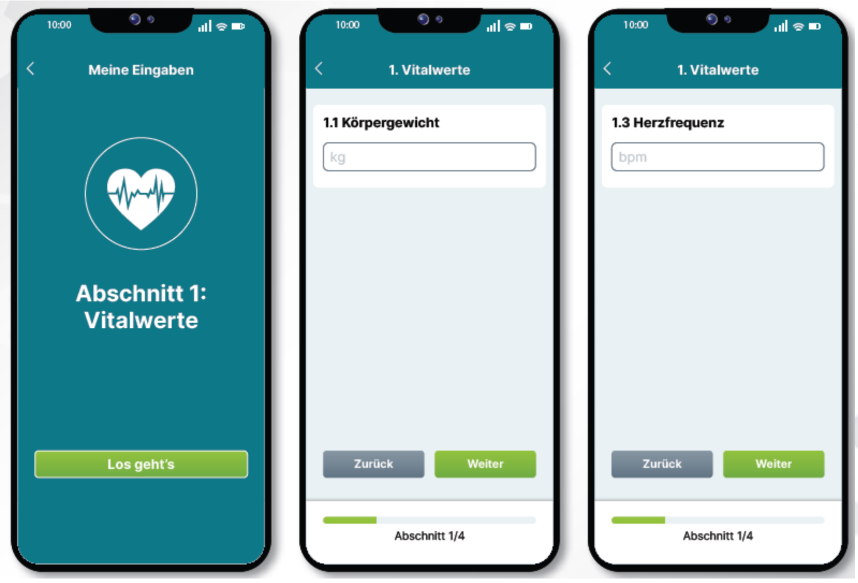

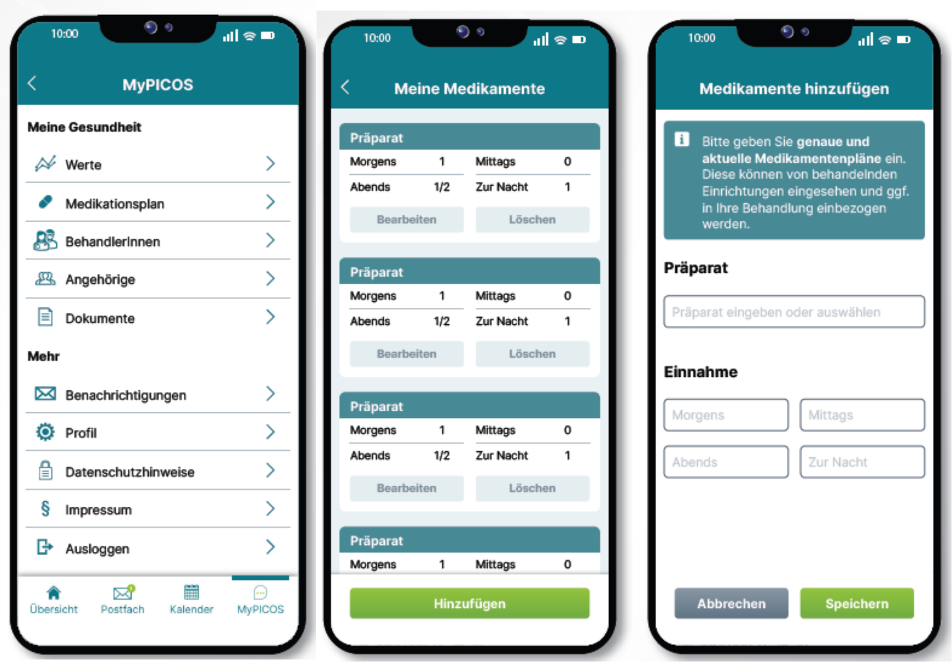
MyPICOS
Meine Gesundheit
Werte
Medikationsplan
BehandlerInnen
Angehörige
Dokumente
Mehr
Benachrichtigungen
Profil
Datenschutzhinweise
Impressum
Ausloggen
Übersicht
Postfach
Kalender
MyPICOS
Meine Medikamente
Präparat
Morgens 1 Mittags 0
Abends 1/2 Zur Nacht 1
Bearbeiten
Löschen
Hinzufügen
Medikamente hinzufügen
Bitte geben Sie genaue und aktuelle Medikamentenpläne ein. Diese können von behandelnden Einrichtungen eingesehen und ggf. in Ihre Behandlung einbezogen werden.
Präparat
Präparat eingeben oder auswählen
Einnahme
Morgens
Mittags
Abends
Zur Nacht
Abbrechen
Speichern

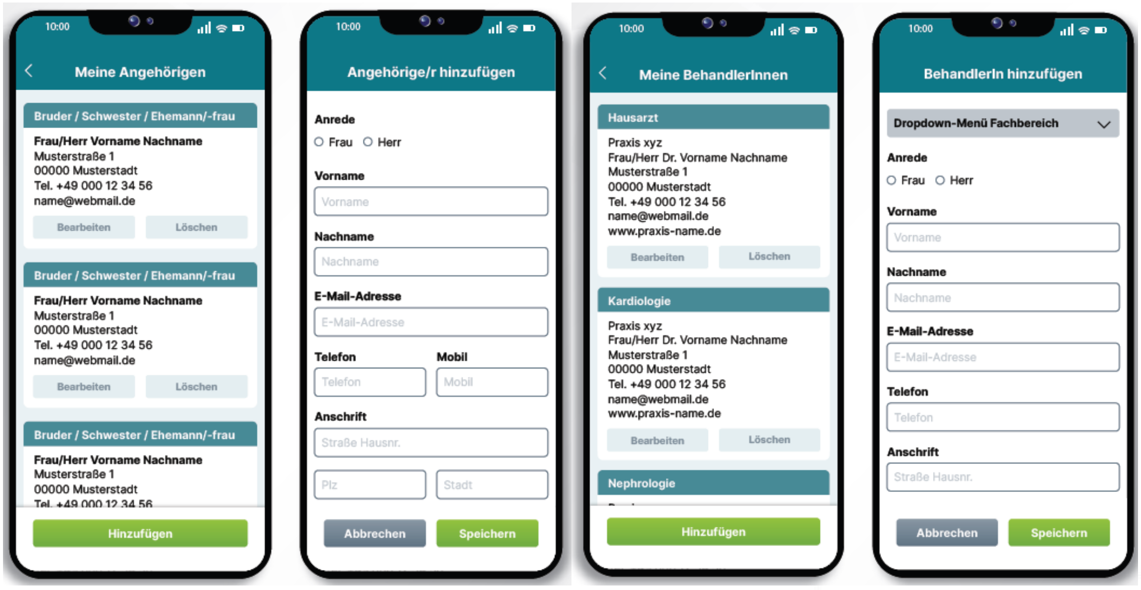
Meine Angehörigen
Bruder / Schwester / Ehemann/-frau
Frau/Herr Vorname Nachname
Musterstraße 1
00000 Musterstadt
Tel. +49 000 12 34 56
name@webmail.de
Bearbeiten
Löschen
Hinzufügen
Angehörige/r hinzufügen
Anrede
Frau
Herr
Vorname
Nachname
E-Mail-Adresse
Telefon
Mobil
Anschrift
Straße Hausnr.
Plz
Stadt
Abbrechen
Speichern
Meine BehandlerInnen
Hausarzt
Praxis xyz
Frau/Herr Dr. Vorname Nachname
Musterstraße 1
00000 Musterstadt
Tel. +49 000 12 34 56
name@webmail.de
www.praxis-name.de
Kardiologie
Nephrologie
BehandlerIn hinzufügen
Dropdown-Menü Fachbereich

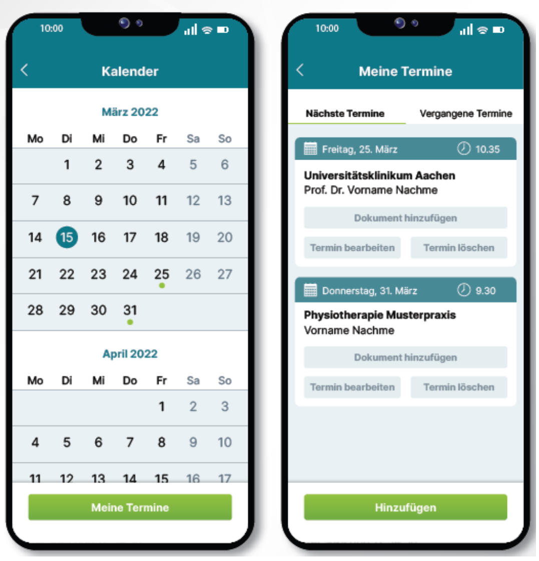
Kalender
März 2022
Mo Di Mi Do Fr Sa So
April 2022
Meine Termine
Nächste Termine
Vergangene Termine
Freitag, 25. März
10.35
Universitätsklinikum Aachen
Prof. Dr. Vorname Nachme
Dokument hinzufügen
Termin bearbeiten
Termin löschen
Donnerstag, 31. März
9.30
Physiotherapie Musterpraxis
Vorname Nachme
Hinzufügen

1. Would you want the support of a relative, caregiver or similar to use the PICOS app?

☐	Yes
☐	No

2. Please indicate the degree to which you agree/disagree with the following statements regarding the PICOS app.

	completely disagree	disagree	slightly disagree	neither agree nor disagree	slightly agree	agree	completely agree
1. Using the PICOS app will be fun.	☐	☐	☐	☐	☐	☐	☐
2. Using the PICOS app will be entertaining.	☐	☐	☐	☐	☐	☐	☐
3. Using the PICOS app will be enjoyable.	☐	☐	☐	☐	☐	☐	☐
4. Using the PICOS app will give me pleasure.	☐	☐	☐	☐	☐	☐	☐
5. Using the PICOS app will be exciting.	☐	☐	☐	☐	☐	☐	☐
6. Using the PICOS app will be thrilling.	☐	☐	☐	☐	☐	☐	☐
7. Using the PICOS app will be delightful.	☐	☐	☐	☐	☐	☐	☐
8. Learning to use the PICOS app will be easy for me.	☐	☐	☐	☐	☐	☐	☐
9. My interaction with the PICOS app will be simple	☐	☐	☐	☐	☐	☐	☐
10. I will find my interaction with the PICOS app clear and understandable.	☐	☐	☐	☐	☐	☐	☐
11. I can easily become skillful at using the PICOS app.	☐	☐	☐	☐	☐	☐	☐

12. It will be easy to remember how to use the PICOS app.	☐	☐	☐	☐	☐	☐	☐
13. Overall, I anticipate that the PICOS app will be easy to use.	☐	☐	☐	☐	☐	☐	☐
14. I will find the PICOS app useful in my daily life.	☐	☐	☐	☐	☐	☐	☐
15. Using the PICOS app can increase my chance of achieving better health.	☐	☐	☐	☐	☐	☐	☐
16. Using the PICOS app helps me to manage (monitor) my health more efficiently.	☐	☐	☐	☐	☐	☐	☐
17. Using the PICOS will increases my ability to take charge of my health.	☐	☐	☐	☐	☐	☐	☐
18. Overall, I would find the PICOS app to be advantageous.	☐	☐	☐	☐	☐	☐	☐
19. Using the PICOS app for health self-management would make me very nervous.	☐	☐	☐	☐	☐	☐	☐
